# The After-Results of Injuries

**Published:** 1908-10-17

**Authors:** 


### The Hospital

A Journal of

The Medical Sciences and Hospital Administration.

New Series. No. 84, Vol. IV. [No. 1156, Vol. XLV.] SATURDAY, OCTOBER 17, 1908.

### The After-results of Injuries.

That peculiar and indescribable form of self-confidence and capacity for dealing adequately with unexpected emergencies, whose disappearance is colloquially called "loss of nerve," is one of the psychic functions about which very little indeed is understood. We know well enough that for many responsible posts are required men not merely of a high degree of skill, but also possessing this subtle quality without which skill is of little use; such are the occupations of captain of a ship, engine-driver, surgeon, detective, scout, and many more. It is, further, common knowledge that the mysterious change which converts a highly valued craftsman into a mediocre one may occur either quite suddenly or so gradually that the process cannot be traced; and it usually happens that in the first alternative some definite cause can be assigned. This may be a physical injury, not necessarily cephalic, or some purely subjective agent such as an emotion. Also, whereas individuals differ very markedly in the extent to which they are endowed with this mental quality, it is broadly true that the more stable his nervous organisation—the more sane the individual, that is—the more of it he is likely to possess. Yet the insane sometimes achieve feats the mere contemplation of which causes sane men a shiver. Of all the effects, immediate and remote, of injury, the psychic are the most difficult accurately to estimate, and the most variable.

In a thoughtful paper read to the Medical Society of London * Mr. Clinton Dent summarises some reflections on this difficult subject suggested by his experience as chief surgeon to the Metropolitan Police Force. His patients, as he points out, are picked men in the prime of life, and they are, to all intents, exempt from occupation diseases. They are, however, especially liable to assault and injury in the execution of their duties, so much so that one-seventh of the whole force sustain lesions of greater or less severity during the course of a year. Now these injuries are sustained very often at moments of great mental tension, as when a constable makes an arrest single-handed in a hostile crowd, or during the chase of some criminal rendered desperate by detection; and even the most trivial of them in appearance may be followed by unlooked-for and severe psychic symptoms. The mere knowledge that at any moment he may be compelled to rush instantly into bodily danger of a sort to which he has already once been exposed with consequent traumata seems to produce "loss of nerve" and even more serious forms of mental instability with surprising frequency. A slight cut, a bad shaking, the loss of a digital phalanx, a broken but well-united bone, all of these seemingly slight accidents sustained when on duty may be followed, after a longer or shorter interval, by disabling loss of nervous control up to complete insanity. Mr. Dent ascribes these peculiar after-results of injury to the very arduous and responsible nature of the police constable's duties. There is for the latter no "soft job" where responsibility is light, such as can be found for the railway pointsman or the sailor; he must go back either to full duty or to none at all, and even the very slightest physical disability will tell unfavourably upon a man who has to spend eight hours a day on his feet in London. The mental condition of the soldier going into action is not comparable to that of a constable arriving at his beat; for the former is encouraged by the proximity of his comrades, and he knows that the ordeal, once over, is not likely to recur soon or without due warning, whereas the latter must thrust himself alone into the hurly-burly the very instant he perceives it. Mr. Dent lays especial stress on the slight accidents associated with subsequent breakdown in his constables, and no doubt it is, as he says, the circumstances surrounding the infliction of an injury which have the greater share in producing these particular after-effects. It would be interesting to know how often a mental strain, such as that experienced in moments of great peril, but unaccompanied by physical injury, is followed by "loss of nerve" sufficient to incapacitate from active service.

Almost equally important are Mr. Dent's observations on the functional results of the repair of certain injuries, particularly fractures. He takes a much less optimistic view of the proportion of complete recoveries after fractures of the leg bones amongst all the labouring classes than do some surgeons, and, as far as the police are concerned, states that he can hardly remember a case of Pott's or Dupuytren's fracture in which recovery has been

* *The Clinical Journal*, Oct. 7th, 1908.

complete enough to enable the man to resume his occupation. There is no doubt that these and other fractures of the leg-bones have sometimes been thought too little of by the medical profession, and it has been often overlooked that the restoration of a leg-bone to a reasonable anatomical copy of what it was previously is very far indeed from implying as complete a functional recovery judged by the wage-earning capacity of the patient.

Of the modern treatment of fractured patella a much brighter report is presented; indeed, it is stated that complete restoration of strength and function, as opposed to almost invariable disability before the days of open operation, can now be confidently anticipated. Of the radical cure of hernia a similar satisfactory picture is drawn. As a contribution to the study of the psychoses following injury the paper may be, as its author calls it, desultory; but it is to be wished that more medical men who enjoy facilities for observing the ultimate effects of disabling injuries in large numbers would record their experiences and impressions in the same way.

